# Supplementary material for: Mapping the spectrum of 3D communities in human chromosome conformation capture data
Source: Sci Rep. 2019 May 2;9:6859. doi: 10.1038/s41598-019-42212-y (PMC6497878; doi:10.1038/s41598-019-42212-y)
Supplement: Supplementary file 1 — Supplementary Information [file 41598_2019_42212_MOESM1_ESM.pdf]

Supplementary Information for “Mapping the spectrum of 3D communities in human chromosome conformation capture data”

Sang Hoon Lee[1,*], **Yeonghoon Kim**[2], **Sungmin Lee**[3], **Xavier Durang**[4], **Per Stenberg**[5], **Jae-Hyung Jeon**[2,†], **and Ludvig Lizana**[6,‡]

[1]Department of Liberal Arts, Gyeongnam National University of Science and Technology, Jinju 52725, Korea
[2]Department of Physics, Pohang University of Science and Technology, Pohang 37673, Korea
[3]Department of Physics, Korea University, Seoul 02841, Korea
[4]Department of Physics, University of Seoul, Seoul 02504, Korea
[5]Department of Ecology and Environmental Science (EMG), Umeå University, Umeå 90187, Sweden
[6]Integrated Science Lab, Department of Physics, Umeå University, Umeå 90187, Sweden
[*]lshlj82@gntech.ac.kr
[†]jeonjh@postech.ac.kr
[‡]ludvig.lizana@umu.se

In this Supplementary Information, we present additional investigations on the polymeric properties of the community subchains in FGs.

Asphericity of the 3D folded structure of community subchains

The asymmetric 3D shape of a polymer chain can be analyzed using the gyration tensor $\mathbf{S}$. For a polymer chain composed of N monomers, the element of the gyration tensor is defined as

$$S_{mn} = \frac{1}{2N^2} \sum_{i=1}^{N} \sum_{j=1}^{N} \left(r_m^{(i)} - r_m^{(j)} \right) \left(r_n^{(i)} - r_n^{(j)} \right) , \tag{1}$$

where $r_n^{(i)}$ is the nth Cartesian coordinate of the monomer $i \in \{1, \cdots, N\}$. The three eigenvalues of the gyration tensor in Eq. (1), denoted by λ_1^2, λ_2^2, and λ_3^2, characterize the overall geometry of the polymer. The radius of gyration, defined in Fig. 1(**f**) in the main text, is obtained as $R_g^2 = S_{xx} + S_{yy} + S_{zz}$ and, using the eigenvalues, given by

$$R_g = \sqrt{\lambda_1^2 + \lambda_2^2 + \lambda_3^2} . \tag{2}$$

The asphericity measuring the relative shape anisotropy is quantified by the parameter κ^2, defined as

$$\kappa^2 = \frac{\left(\lambda_1^2 - \lambda_2^2\right)^2 + \left(\lambda_2^2 - \lambda_3^2\right)^2 + \left(\lambda_3^2 - \lambda_1^2\right)^2}{2\left(\lambda_1^2 + \lambda_2^2 + \lambda_3^2\right)^2} = \frac{3\left(\lambda_1^4 + \lambda_2^4 + \lambda_3^4\right)}{2\left(\lambda_1^2 + \lambda_2^2 + \lambda_3^2\right)^2} - \frac{1}{2} . \tag{3}$$

κ^2 varies from 0 (isotropic objects having the uniform eigenvalues) to 1 (severely elongated objects having a single dominant eigenvalue). In Supplementary Fig. S1, we plot the asphericity κ^2 as a function of subchain length for the communities and randomly chosen subchains in fractal and equilibrium globules. For an ideal unconfined chain (dashed-dotted line), the asphericity is known to be $\kappa^2 \simeq 0.39$[1]. When for FGs and EGs the subchain length is too short to feel the confinement, κ^2 for the subchains has roughly this ideal value, and decreases with the chain length. We find that, compared to the average subchains in FGs, the communities therein have smaller κ^2 values, thus being more sphere-like than the FG itself. Together with the end-to-end distance analysis in Fig. 1 in the main text, this indicates that the community subchains in FGs have a 3D structure where both ends (i.e., the boundary sites in its contact map) are close to each other and the monomer distribution is almost isotropic. The reference system (EGs) shows a similar trend while the communities in FGs are more sphere-like than in EGs up to the subchain length ~ 500. Beyond this length, κ^2 for EGs approaches zero for the randomly chosen subchains, due to the finite-size effect caused by the simulation protocol for making an EG using the random walk inside a sphere.

The results for all of the chromosomes

In Supplementary Figs. S2–S5, for all of the chromosomes, we show the average coverage of RNA-seq reads within communities for different γ values.

Chromatin state analysis

In Supplementary Fig. S6, we use the chromatin states to investigate what types of chromatin states are enriched in different communities at different γ values.

References

1. Rudnick, J. & Gaspari, G. The shapes of random walks. *Science* **237**, 384–389, DOI: 10.1126/science.237.4813.384 (1987). https://doi.org/10.1126/science.237.4813.384.
2. Ernst, J. & Kellis, M. Discovery and characterization of chromatin states for systematic annotation of the human genome. *Nat. Biotechnol.* **28**, 817–825 (2010).
3. Ernst, J. *et al.* Mapping and analysis of chromatin state dynamics in nine human cell types. *Nature* **473**, 43–49 (2011).

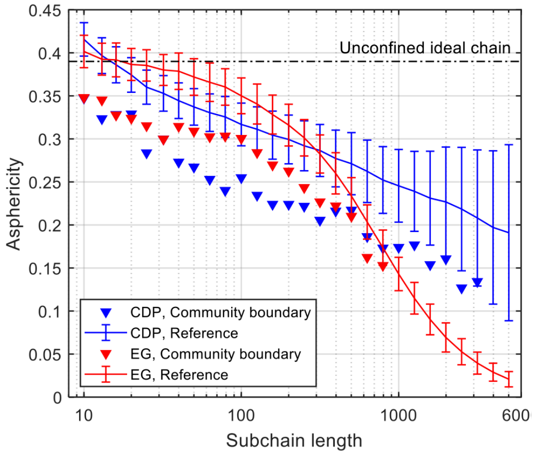

Supplementary Figure S1. The asphericity κ^2 as a function of subchain length for fractal (FG, blue) and equilibrium globules (EG, red) averaged over 200 polymer realizations. The triangles denote the asphericity for the communities, and solid lines the polymer as a whole. To find the communities, we use $\gamma = 0.4$. The data were obtained from the simulation of 200 sample globules for each polymer model. The error bars show the standard error of the mean. The dashed-dotted horizontal line corresponds to the value ≈ 0.39 for unconfined ideal chain[1].

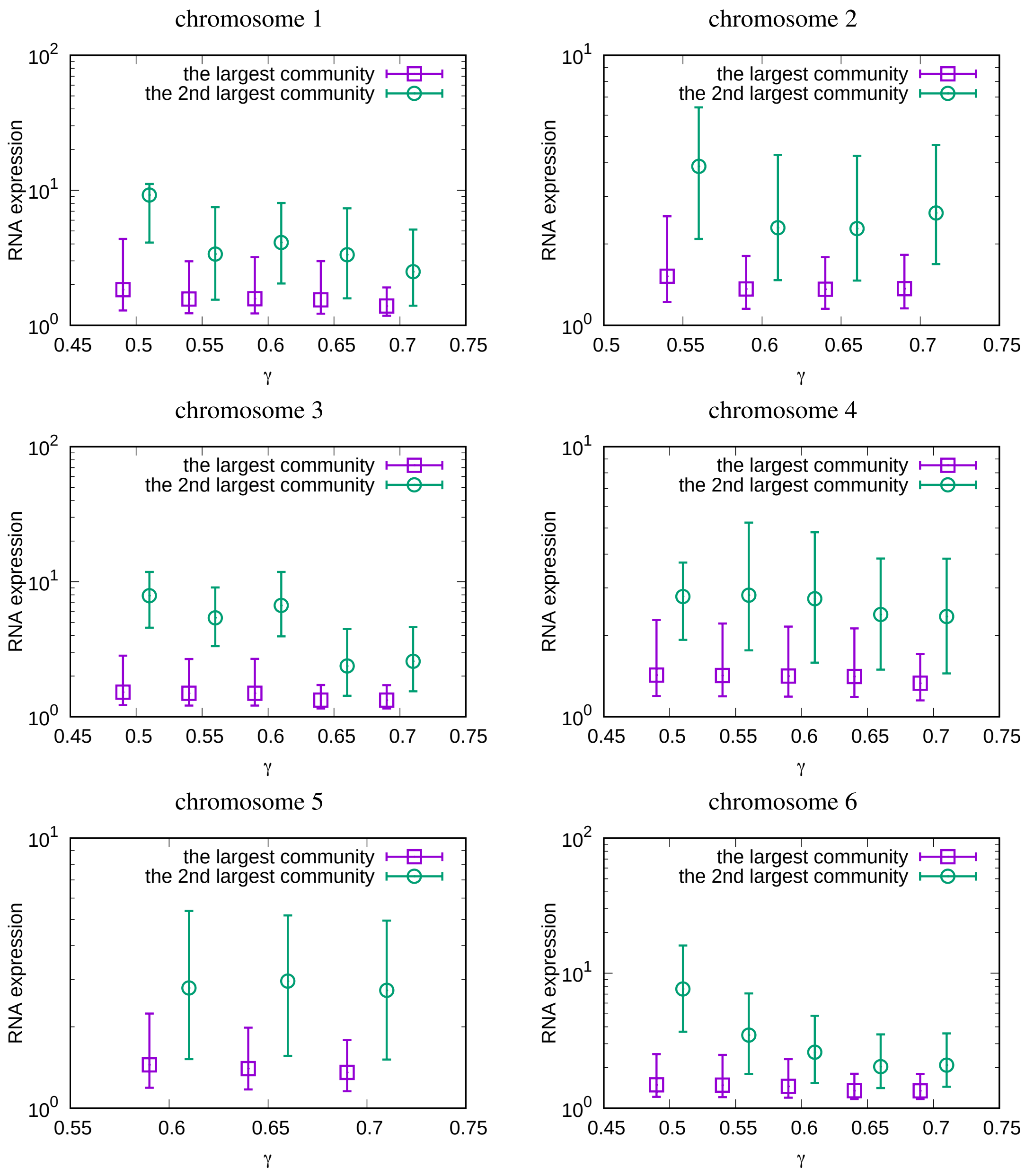

Supplementary Figure S2. Differential RNA expression levels for different communities. We present the differential RNA expression for communities corresponding to various values of γ for chromosomes 1–6, only for the largest and second largest communities. For visualization, we shift the data points slightly to the left for the largest community and right for the second largest community for each of the γ values. The plots indicate the median values with the quartiles as the error bars.

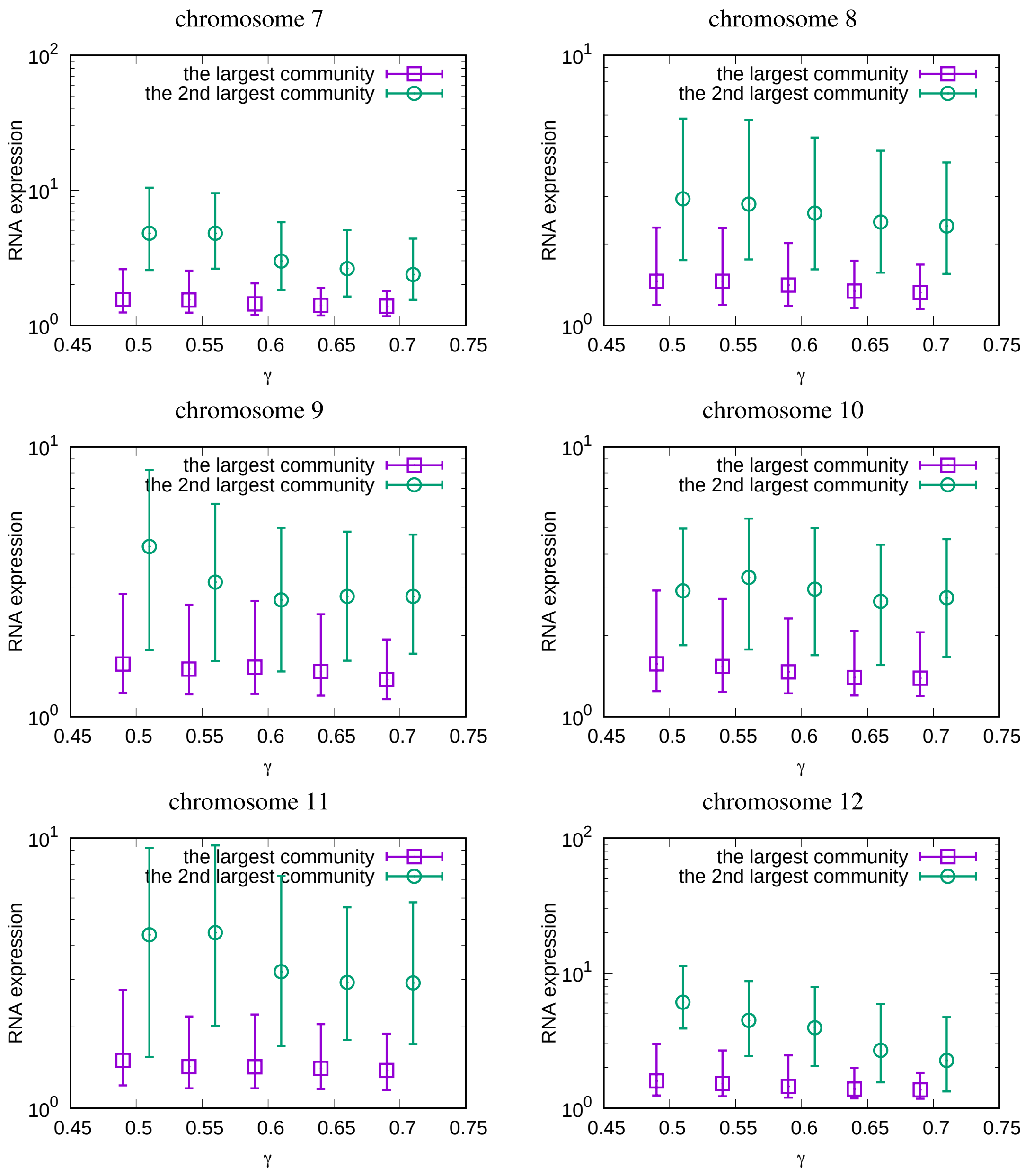

Supplementary Figure S3. Differential RNA expression levels for different communities. We present the differential RNA expression for communities corresponding to various values of γ for chromosomes 7–12, only for the largest and second largest communities. For visualization, we shift the data points slightly to the left for the largest community and right for the second largest community for each of the γ values. The plots indicate the median values with the quartiles as the error bars.

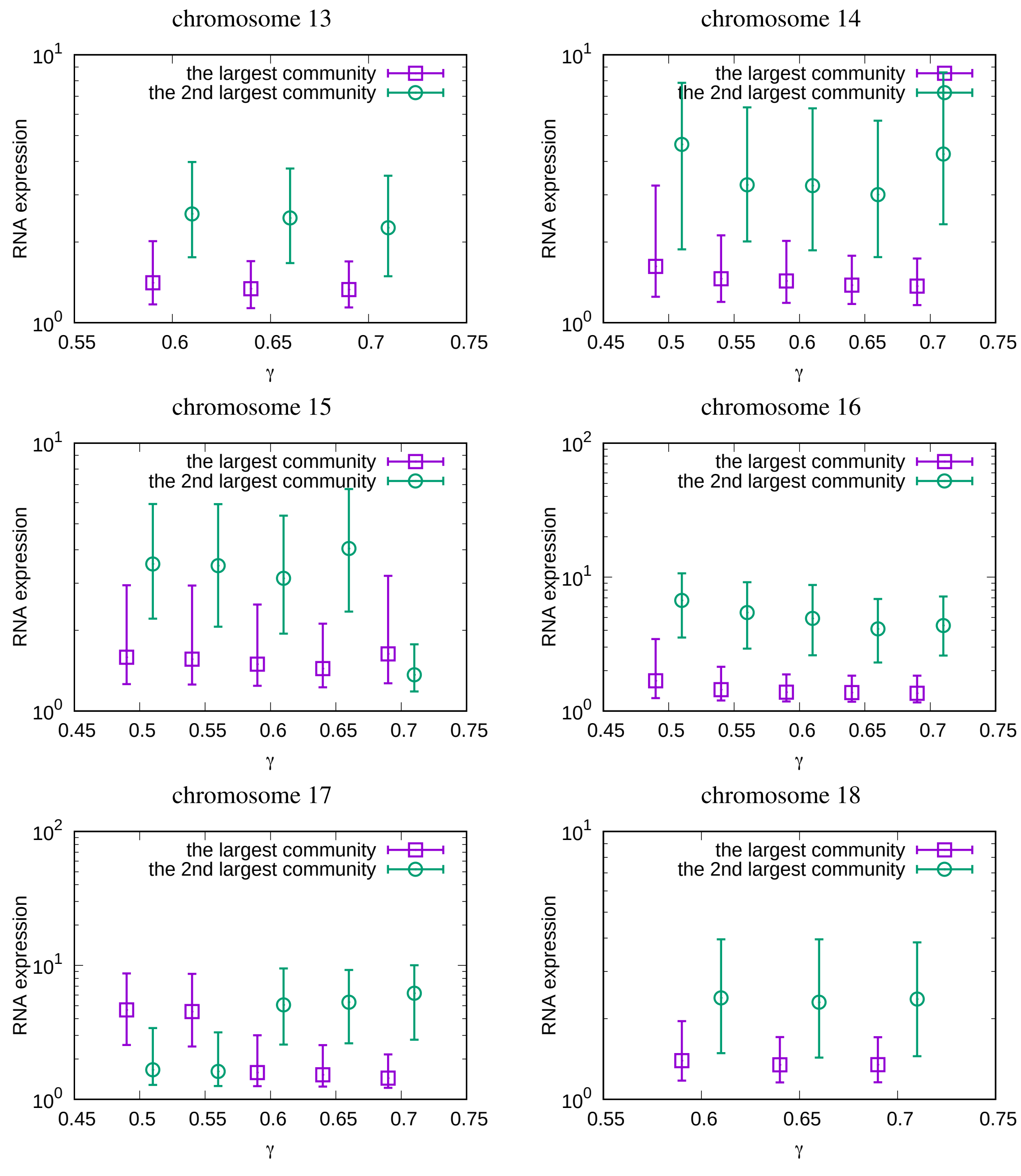

Supplementary Figure S4. Differential RNA expression levels for different communities. We present the differential RNA expression for communities corresponding to various values of γ for chromosomes 13–18, only for the largest and second largest communities. For visualization, we shift the data points slightly to the left for the largest community and right for the second largest community for each of the γ values. The plots indicate the median values with the quartiles as the error bars.

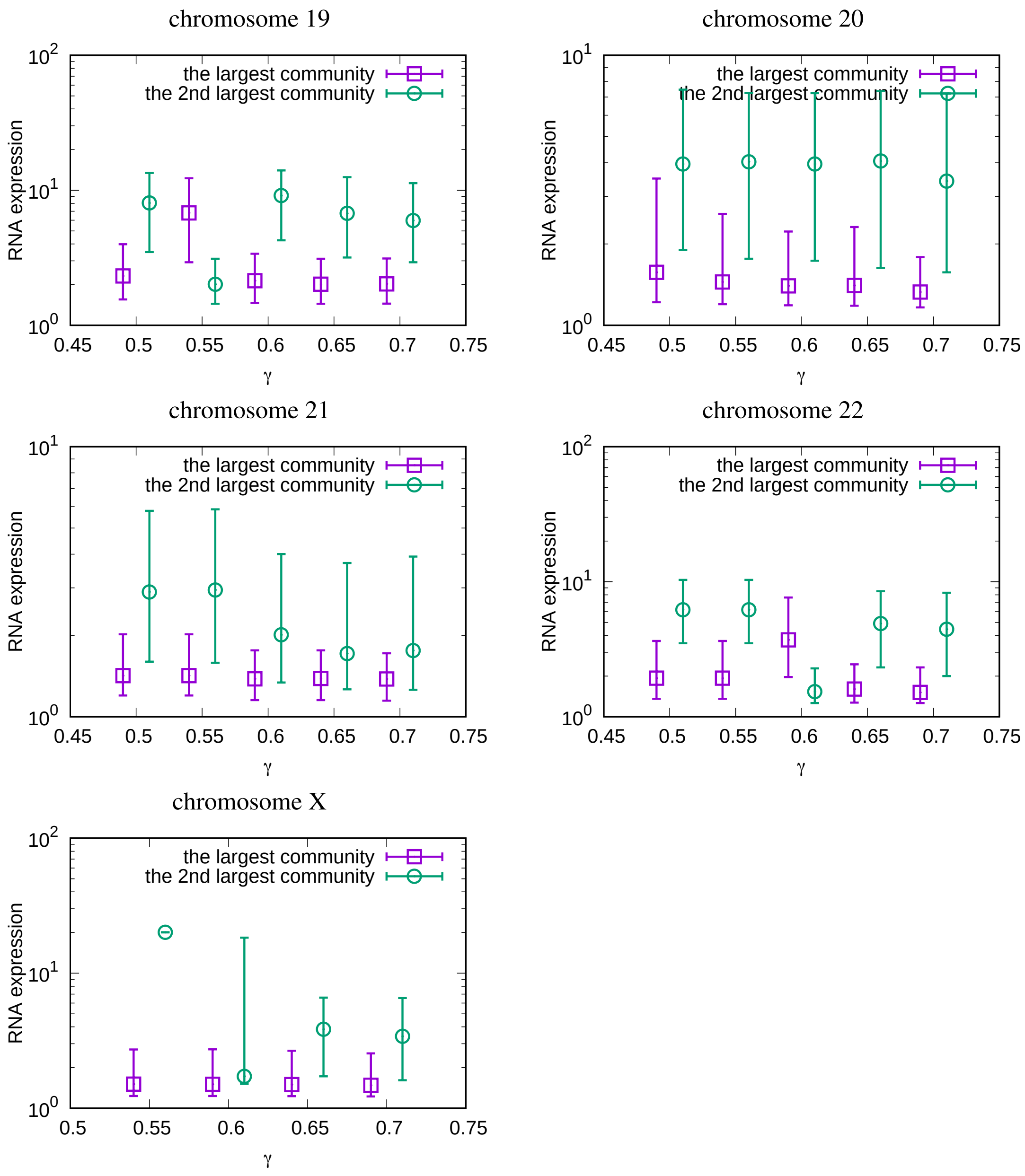

Supplementary Figure S5. Differential RNA expression levels for different communities. We present the differential RNA expression for communities corresponding to various values of γ for chromosomes 19–22 and X, only for the largest and second largest communities. For visualization, we shift the data points slightly to the left for the largest community and right for the second largest community for each of the γ values. The plots indicate the median values with the quartiles as the error bars.

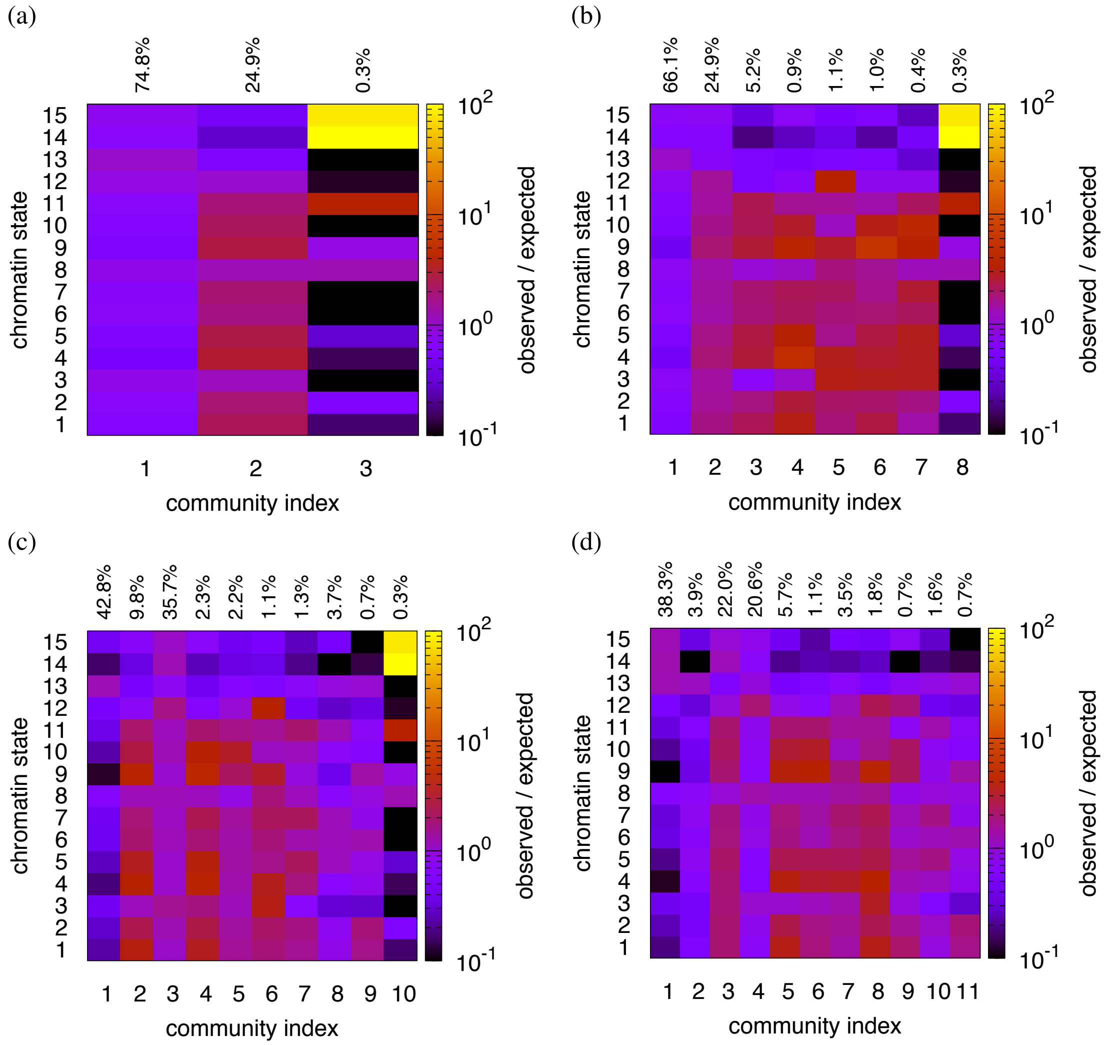

Supplementary Figure S6. The composition of chromatin segments for each community found for chromosome 1, in terms of chromatin states[2,3]. The color bar represents the fold difference between the total length of overlapping chromatin segments (in the unit of sequence) between the given community and the given chromatin state, and the expected overlap by assuming the random pairing between the communities and chromatin states. The percentage above each community index indicates the relative fraction of the community in the sequence. For all of the cases, $\chi^2 > 5 \times 10^7$ and the result is statistically significant with p-value $< 10^{-5}$. The values of the resolution parameter are (a) $\gamma = 0.6$, (b) $\gamma = 0.65$, (c) $\gamma = 0.7$, and (d) $\gamma = 0.75$. The chromatin states are 1: active promoter, 2: weak promoter, 3: poised promoter, 4: strong enhancer, 5: strong enhancer, 6: weak enhancer, 7: weak enhancer, 8: insulator, 9: transcriptional transition, 10: transcriptional elongation, 11: weak transcribed, 12: polycomb-repressed, 13: heterchromatin; low signal, 14: repetitive/copy number variation, and 15: repetitive/copy number variation.
